# An Evaluation on the Effect of Health Education and of Low-Dose Statin in Dyslipidemia among Low-Income Rural Uyghur Adults in Far Western China: A Comprehensive Intervention Study

**DOI:** 10.3390/ijerph120911410

**Published:** 2015-09-11

**Authors:** Jiaolong Ma, Shuxia Guo, Rulin Ma, Jingyu Zhang, Jiaming Liu, Yusong Ding, Mei Zhang, Heng Guo, Jia He, Yizhong Yan, Lati Mu, Shugang Li, Qiang Niu

**Affiliations:** Key Laboratory of Xinjiang Endemic and Ethnic Diseases, School of Medicine, Shihezi University, Beier Road, Shihezi City, Xinjiang Uyghur Autonomous Region 832000, China; E-Mails: jiaojiaolong881202@163.com (J.M.); marulin@126.com (R.M.); yfyxxzjy@126.com (J.Z.); liujiaming@shzu.edu.cn (J.L.); 13399931625@163.com (Y.D.); zmberry@foxmail.com (M.Z.); guoheng@shzu.edu.cn (H.G.); hejia123.shihezi@163.com (J.H.); erniu19880215@sina.com (Y.Y.); murat08123@163.com (L.M.); lishugang@ymail.com (S.L.); niuqiang1214@163.com (Q.N.)

**Keywords:** dyslipidemia, comprehensive intervention, rural, evaluation, Uyghur

## Abstract

*Objective*: To evaluate the effect of comprehensive intervention by health education and medical intervention to dyslipidemia Uyghur patients in low-income rural areas in Xinjiang, China. *Method*: A multistaged (prefecture-county-township-village) stratified cluster random sampling method was used to select participants in southern Xinjiang. Twelve villages in Jiangbazi Township in Jiashi County were chosen. These villages were randomly divided into six intervention groups and six control groups, and local Uyghur aged 18 years or older residing in the village for at least 6 months were interviewed for a baseline prevalence study and to select participants for two years of comprehensive intervention including low dose simvastatin and the effects of the interventions were observed. *Results*: A total of 655 participants (347 participants in the intervention groups, 308 participants in the control groups) were randomly selected from 12 villages in Jiangbazi Township, follow-up rate is 87.0%. Compared to baseline mean levels of TG and LDL-C were reduced by 1.39 mmol/L (*p* < 0.01) and 2.12 mmol/L (*p* < 0.01) respectively and levels of HDL-C increased by1.22 mmol/L (*p* < 0.01) in the intervention group. Lipids were controlled in 38.61% of the intervention groups *vs.* 3.57% of the control groups (*p* < 0.01). Compared with baseline lipid levels, TG, TC, LDL-C and HDL-C was significantly improved, compared with it was in control groups. *Conclusions*: Blood lipid levels of Uygur patients with dyslipidemia can be effectively improved through health education combined with low-dose statin administration. This suggests that national strategies in public health be developed to improve the treatments to low-income rural minorities with dyslipidemia.

## 1. Introduction

Cardiovascular disease (CVD) is one of the major contributions to mortality in developed and developing countries, especially in low- and middle-income countries [1,2,3]. The World Health Organization has estimated that cardiovascular disease (CVD) accounted for more than 30% of deaths around the world in 2008, and almost 25 million people are expected to die of cardiovascular disease in 2030 [4]. In China with the economic growth and associated lifestyle changes, people's living standards have advanced. Numerous studies have shown that the prevalence of dyslipidemia was relatively high during the last decade, and the percentage of adults with awareness, treatment and control of dyslipidemia were low in China [5,6,7,8]. However we believe that cardiovascular-related death could be prevented with adoption and implementation of appropriate treatment and preventive measures [9].

Xinjiang is a multi-ethnic region in the northwest of the People’s Republic of China, which is the largest Chinese administrative division and spans over 1.6 million km^2^, thus occupying about one sixth of the country’s territory. The Uyghur is one of the largest inhabitant minority groups in Xinjiang Uyghur Autonomous Region, located about 4407 km (2739 miles) away from Beijing. There were approximately 19.63 million residents living in this beautiful place and among them the Uygur accounted for 45.73% of the population (data from 5th census of China). On the one hand, with relatively underdeveloped economic and limited health resources, there have not been serious studies to analyze local public health needs including long-term monitoring for patients with dyslipidemia and provide patients with treatment which is appropriated to their needs. On the other hand, due to differences in religion, culture, lifestyle, diet, and genetic background in Uyghur, effective and practicable comprehensive interventions for patients might have a positive meaning for reducing associated complications and improved the health in preventive public health for inhabitant residents in Xinjiang.

Several studies have reported dyslipidemia is highly prevalent in Xinjiang, and the proportion of participants with dyslipidemia who are aware of, treated, and controlled is unacceptably low [10,11,12]. To better help dyslipidemia patients control blood lipid levels, our researchers performed regular health education and provided lipid-lowering drugs to adjust the lipid levels for local dyslipidemia patients, and raised awareness, treatment and control of dyslipidemia between May 2009 and November 2012, which have important implications in preventive public health for medically underserved Muslim minorities.

## 2. Methods

### 2.1. Ethics Statement

Ethics approval was obtained from the Ethics Committee of the First Affiliated Hospital of Shihezi University School (IERB No. SHZ2010LL01). Informed consent, voluntary participation, confidentiality, and anonymity followed standard University hospital guidelines. All participants gave written informed consent before the study began.

### 2.2. Study Design and Patients

A community-based, ethnic minority in rural areas, comprehensive intervention study involved in regulating blood lipids for dyslipidemia patients was conducted from 2009 to 2012 in Xinjiang (Kashi). A multistage (prefecture-county-township-village) stratified cluster random sampling method was used to select participants. First of all, according to geographical distributions of the minority populations in Xinjiang, we randomly elected one county in each prefecture and one township from each county (Jiangbazi Township in Jiashi County). Secondly, 12 villages in Jiangbazi Township were selected to represent the Muslim Uyghur in rural areas. These villages were then randomly divided into six intervention groups and six control groups, and we interviewed local Uyghur residents aged 18 years or older in the villages over six months to conduct a baseline prevalence survey. We successfully interviewed a total of 3625 Uygur individuals in the baseline survey, and the overall prevalence rates of dyslipidemia in Uygur adults were 42.4%. Long-term emigrants, fluid population and unwilling subjects were excluded. At the last stage, 753 individuals were selected (397 participants in the intervention groups, 356 participants in the control groups). After a three-year comprehensive intervention, we successfully followed up a total of 655 individuals (347 participants in the intervention groups, 308 participants in the control groups), follow-up rate was 87.0% (87.4% in the intervention groups and 86.5% in the control groups).

### 2.3. Interventions

**Figure 1:**
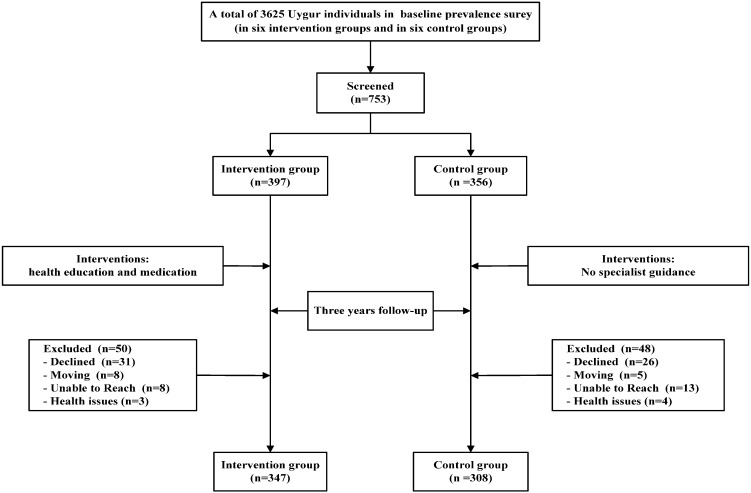
Patient selection and randomization.

In the whole intervention process, health education and guidance for statin treatment were offered to participants for free during face-to-face interviews. Health education intervention has been regularly provided to strengthen the intervention, including the publicized basics of dyslipidemia (low-fat and less-salt diet, healthy lifestyle, *etc.*), and Uyghur language materials (calendars, magazines, brochures, *etc.*) distributed using local translators. We started the baseline survey in May 2009 to choose the participants with the first health education. The first follow-up began in November 2009, followed up at the end of each year and three times in total. To regularly give intensive interventions, we conducted semiannually dyslipidemia prevention lectures which were at least 45 min, a total of seven times. For the intervention groups, statins (simvastatin) were provided to participants for free, and the starting dose was 10 mg, once a day by oral administration. During follow-up period, the dose should be adjusted for during four weeks or more when we monitored lipid levels, the maximum could be gradually increased to 40 mg. Health education and drug instructions were used to explain to treatment to the participants in the local language. The control groups was a general advocacy groups which didn`t receive specialist guidance. See Figure 1 for the flow chart describing the number of participants at each stage.

### 2.4. Data Collection

#### 2.4.1. Questionnaire Survey

A self-developed questionnaire was applied to collect detailed information from all respondents during face-to-face interview. A questionnaire which we developed was applied demographic information on respondents (such as age, gender, address, education level, and marital status, *etc.*), personal lifestyles (such as smoking, alcohol intake, and dietary habits, *etc.*), the follow-up questionnaire (such as Basic Information, code, ID number, lifestyle behaviors, salt intake, symptoms, medication and the amount of medication, adverse drug reactions, *etc.*).

#### 2.4.2. Physical Examination and Blood Lipid Examination

In the interview and physical examinations, anthropometric measurements of height, weight, waist circumference and hip circumference were obtained using a standard protocol for participants. WC was defined as the midpoint between the lower rib and upper margin of the iliac crest, measured by a ruler tape with an insertion buckle at one end. WC was measured to the nearest 0.1 cm. During the interview, a 5-mL fasting blood sample (fasting 8 h) was collected in an EDTA vacutainer tube. Serum was separated from the samples within 10 min immediately and stored at −80 °C. We measured the concentrations of triglycerides (TG), total cholesterol (TC), high density lipoprotein-cholesterol (HDL-C), and low density lipoprotein-cholesterol (LDL-C) using an automated biochemical instrument (OLYMPUS AU2700, Tokyo, Japan) in the Clinical Laboratory Department of the First Affiliated Hospital of Shihezi University School.

#### 2.4.3. Definitions

The definition of dyslipidemia was based on the guideline of Chinese Prevention and Treatment of Dyslipidemia in adults. Thus, subjects were considered as dyslipidemic if their total cholesterol ≥6.22 mmol/L (240 mg/dL) and/or his LDL-C ≥ 4.14 mmol/L (160 mg/dL) and/or HDL-C < 1.04mmol/L (40 mg/dL), and/or triglyceride ≥ 2.26 mmol/L (200 mg/dL) and/or they were taking a lipid lowering drug. In addition we excluded those who had active hepatitis or persistent elevated serum aminotransferase, severe heart or brain disease, cerebral vascular disease, cancer, pregnancy and breast-feeding women, those who are allergic to any ingredient and reluctant to participate in research or not able to participate in follow-up. Participants were considered to have controlled dyslipidemia if concentrations were TC < 6.22 mmol/L, LDL-C < 4.14 mmol/L, HDL-C ≥ 1.04 mmol/L, and TG < 2.26 mmol/L after treatment.

#### 2.4.4. Statistical Analysis

All of the questionnaire data were double-entered and cross validated using EpiData version 3.1 (EpiData Association, Odense, Denmark). Data were then analyzed using SPSS (Statistical Program for Social Sciences, version 20.0, IBM, Armonk, NY, USA). Continuous variables were given as mean ± standard deviation and analyzed using t-test. Categorical variables were expressed as numbers or percentages and analyzed using the Chi-square test. Baseline, follow-up and outcomes period lipid levels were compared using repeated measures data of ANOVA. All statistical tests were two-sided and differences were considered statistically significant when *p* value 0.05.

## 3. Result

### 3.1. Basic Characteristics of the Study Population

Characteristics of the study participants enrolled in this study, as stratified by groups, are shown in Table 1. A total of 655 participants (326 men and 329 women) were included in this study. Mean ages of the subjects were 45.9 and 45.4 years for the intervention and control groups, respectively. Total follow-up rate was 87.0%. In the intervention groups, there were 397 participants in the baseline and 347 participants in the outcome, follow-up rate was 86.7%. In the control groups, there were 356 participants at baseline and 308 participants in the outcome, follow-up rate was 86.5%. At baseline no significant differences were found between two groups (*p* > 0.05).

**Table 1:** Basic characteristics of the study population at baseline.

Variables	Intervention Group (*n* = 347)	Control Group (*n* = 308)	t/*χ^2^*	*p*
Sex (n,%)			2.118	0.146
male	182(52.4%)	144(46.8%)		
female	165(47.6%)	164(53.2%)		
Age (years)	45.86 ± 15.32	45.37 ± 17.55	0.346	0.729
Height (cm)	163.44 ± 6.99	162.80 ± 10.34	0.187	0.853
Weight (kg)	65.46 ± 10.78	66.28 ± 11.91	1.803	0.093
WHR	0.85 ± 0.06	0.86 ± 0.07	1.841	0.066
BMI (kg/m^2^)	21.51 ± 2.61	21.54 ± 2.83	0.132	0.895
TG (mmol/L)	1.43 ± 0.78	1.29 ± 0.73	1.933	0.054
TC ( mmol/L)	4.39 ± 1.05	4.15 ± 1.20	2.891	0.089
HDL-C ( mmol/L)	0.96 ± 0.23	1.02 ± 0.22	1.796	0.073
LDL-C (mmol/L)	2.44 ± 0.69	2.39 ± 0.86	0.634	0.526
SBP (mmHg)	129.28 ± 22.73	130.76 ± 21.16	0.752	0.457
DBP (mmHg)	80.94 ± 13.79	81.00 ± 14.19	0.043	0.966
FBG (mmol/L)	4.23 ± 0.73	4.09 ± 0.78	1.695	0.091
Current smoking state (%)			1.345	0.510
no	323(93.1%)	295(95.8%)		
yes	24(6.9%)	13(4.2%)		
Current drinking state (%)			1.052	0.591
no	342(98.6%)	304(98.7%)		
yes	5(1.4%)	4(1.3%)		

Data are expressed as means ± SD or *n* (%). Note: BMI: body mass index; WHR, Waist-to-hip ratio; TC: total cholesterol; HDL-C: high-density lipoprotein cholesterol; LDL-C: low-density lipoprotein cholesterol; TG: triglyceride; SBP: systolic blood pressure; DBP: dystolic blood pressure; FBG: fasting blood glucose.

### 3.2. Comparison of Lipid Levels

Variations of abnormal lipid levels by groups at different time points are presented in Table 2. Compared to baseline, levels of TG, LDL-C patients in the intervention groups were reduced and levels of HDL-C were significantly increased *(p* < 0.01).

**Table 2:** Comparison of the survey population lipid levels at different time points.

Variable		Baseline	Post-1 Year	Post-2 Year	Outcome	*F*	*p*
Intervention group	TG	1.43 ± 0.78	1.42 ± 1.03	1.40 ± 1.11 *	1.39 ± 0.81 *^,##^	25.611	0.000
	TC	4.39 ± 1.05	4.22 ± 0.94 **^##^**	4.10 ± 0.83 **^##^**	4.15 ± 0.92 **^##^**	0.603	0.547
	HDL-C	0.96 ± 0.23	1.01 ± 0.37 **	1.09 ± 0.25 *	1.22 ± 0.41 *^,##^	76.877	0.000
	LDL-C	2.44 ± 0.69	2.37 ± 0.59 **^,**##**^	2.10 ± 0.50 *^,#^	2.12 ± 0.62 *^,#^	98.453	0.000
Control group	TG	1.29 ± 0.73	1.37 ± 0.85 **	1.41 ± 0.72 **	1.50 ± 0.91 *	22.493	0.000
	TC	4.15 ± 0.72	4.01 ± 0.92	3.80 ± 0.86	3.95 ± 0.84	0.412	0.703
	HDL-C	1.02 ± 0.22	0.93 ± 0.32 **	1.06 ± 0.29 **	1.10 ± 0.36 **	2.385	0.032
	LDL-C	2.39 ± 0.86	2.43 ± 0.81 **	2.32 ± 0.70 **	2.24 ± 0.77 **	1.754	0.041

All values are mean ± standard deviation (SD) unless otherwise specified. Note: post-1 year, post-2 year and outcomes were compared with baseline, respectively. * *p* < 0.01, ** *p* < 0.05; between-group comparison of baseline TG, TC, HDL-C, LDL-C levels, **^#^** *p* < 0.01, **^##^** *p* < 0.05.

Levels of TG, HDL-C patients in the control groups were increased from baseline to outcome (*p* < 0.01) while levels of LDL-C was reduced (*p* < 0.05). Levels of TG, TC, LDL-C and HDL-C which were the primary outcome variables were significantly better in intervention compared with control groups.

### 3.3. Comparison of the Control Rate of Dyslipidemia

Table 3 shows that after a comprehensive intervention, the percentage of control of dyslipidemia, intervention groups was significantly higher than control groups (38.61% *vs.* 3.57%, *p* < 0.001). There are about 53% of the patients using simvastatin 10 mg/day, 38% of the patients have adjusted to 20 mg/day, only 9% patients have using simvastatin 40 mg/day. There were 347 patients in intervention groups, after lipid-lowering therapy, 38.61% of this population have been controlled to normal, however, only eleven among 308 patients in control groups arrived to the goals.

**Table 3:** The control rate of dyslipidemia in the intervention and control groups.

Groups	*n*	Control	%	*χ^2^*	*p*
Intervention Group	347	134	38.61	116.263	0.000
Control Group	308	11	3.57		

Data are expressed as *n* (%).

### 3.4. Lipid Changes in the Amplitude of the Survey Population

Changes in regard to different lipid components are presented in Table 4. After comprehensive intervention for three years, the percent change from the magnitude in TG was significantly better with intervention groups compared with control groups (−2.80% *vs.* 16.28%, *p* < 0.01). The mean percent change in TC levels was −5.47% in the intervention groups, compared with −4.55% in the control groups (*p* < 0.01).

**Table 4:** Percent change from lipid components between baseline and outcome from intervention and control groups.

Variable		Intervention Group (*n* = 347)	Mean Percent Change, % ± SE (Median)	Control Group (*n* = 308)	Mean Percent Change, % ± SE (Median)
TG, mmol/L	Baseline	1.43 ± 0.78	−2.80 ± 0.08 (0.55)	1.29 ± 0.73	16.28 ± 0.52 (10.99)
	Outcome	1.39 ± 0.81		1.50 ± 0.91 **	
TC, mmol/L	Baseline	4.39 ± 1.05	−5.47 ± 0.09 (−3.81)	4.40 ± 0.12	−4.55 ± 0.19 (−2.16)
	Outcome	4.15 ± 0.92		4.20 ± 0.84 **	
HDL-C, mmol/L	Baseline	0.96 ± 0.23	27.08 ± 0.07 (17.40)	1.02 ± 0.22	7.84 ± 0.02 (6.32)
	Outcome	1.22 ± 0.41		1.10 ± 0.36 **	
LDL-C, mmol/L	Baseline	2.44 ± 0.69	−13.11 ± 0.20 (−9.26)	2.43 ± 0.86	−6.17 ± 0.06 (−6.57)
	Outcome	2.12 ± 0.62		2.28 ± 0.77 *	

All values are mean ± standard deviation (SD) unless otherwise specified. Note: *p* values indicate the percent change between the two groups.* *p* < 0.05, ** *p* < 0.01, between-group comparison of baseline TG, TC, HDL-C, LDL-C levels.

The intervention groups were associated with a mean percent change in HDL-C of 27.08% after three-year treatment, compared with 7.84% in the control groups(p < 0.01).The mean percent change in LDL-C levels was −13.11% in the intervention groups and −6.17% in the control groups in the end of study (p < 0.01).

### 3.5. Comparison of Body Mass Index

At baseline no significant differences were found between two groups (22.55 ± 2.67 kg/m^2^ *vs.* 22.17 ± 2.70 kg/m^2^, *p* > 0.05). After three-year treatment, variations of BMI in intervention groups were significantly lower than control groups (21.86 ± 2.74 *vs.* 23.95 ± 2.54, *p* < 0.05).

### 3.6. Comparison of Knowledge, Attitude, Behavior Score

Table 5 displays comparison of the knowledge, attitude and behavior score between baseline and outcome from intervention and control groups. After three-year comprehensive interventions, the intervention group were significantly higher than control group (*p* < 0.01).

**Table 5:** Comparison of the knowledge, attitude and behavior score between baseline and outcome from intervention and control groups ($\bar{x}$ ± s).

Scores	Intervention Group		Control Group	
	Baseline	Outcome	Baseline	Outcome
Knowledge scores	1.00 ± 1.19 *	3.32 ± 1.29 **^#^**	1.05 ± 1.15	1.39 ± 1.18
Attitude scores	0.60 ± 0.73 *	2.07 ± 0.87 **^#^**	0.66 ± 0.80	0.82 ± 0.92
Behavior scores	0.45 ± 0.71 *	1.11 ± 0.83 **^#^**	0.50 ± 0.81	0.70 ± 0.96
Total average scores	2.05 ± 1.82 *	6.50 ± 3.30 **^#^**	2.20 ± 1.92	2.91 ± 2.31 *

All values are mean ± standard deviation (SD) unless otherwise specified. Note: *p* values indicate Intervention group and control group were compared in the same period, **^#^** *p* < 0.01, **^##^** *p* < 0.05; compare intervention group and control group in the same group, * *p* < 0.01.

## 4. Discussion

Dyslipidemia is an occult chronic disease, and it is one of the major risk factors of cardiovascular disease. Studies reported that the prevalence of dyslipidemia has gradually increased in Chinese adults with rapid economic growth and lifestyle changes [13,14,15]. The objective of our study population was in a place in the remote minority areas in southern of Xinjiang where many studies have shown a higher prevalence of dyslipidemia than the national average [16,17], due to limited resources in public health and an unacceptably low sense of self-care and a rate of awareness, treatment, and control of dyslipidemia is significantly lower than elsewhere. Few of them took targeted interventions to lower lipids and most ignored the high impact of chronic disease on them.

In our study, a comprehensive intervention which combined health education with lipid lowering drugs was carried out in rural Uyghur adults in the northwest. After lipid-lowering treatment in the intervention groups, the average levels of TG, TC and LDL-C were reduced (1.39 mmol/L, 4.15 mmol/L, 2.12 mmol/L), while was lower than local Han adults (1.62 mmol/L, 4.60 mmol/L, 2.23 mmol/L) [11]. The average level of HDL-C has been raised from 1.00 mmol/L to 1.22 mmol/L, which was significantly higher than the level of local Uyghur adults (1.16 mmol/L) [13]. Zhang JD *et al.* [18] reported that 345 patients with dyslipidemia were implemented with health management in the Jinsong district of Beijing. After the one-year intervention, the average levels of TG, TC, HDL-C and LDL-C were respectively 2.0 mmol/L, 5.4 mmol/L, 1.3 mmol/L and 3.1 mmol/L. The MEGA Study evaluated the effect of low-dose pravastatin on primary prevention of CVD in 7832 Japanese patients followed for more than five years, they found that statins reduced lipid levels to a greater degree in diet plus pravastatin than in diet alone [19]. To sum up the results obtained, we found that by reducing the salt with spoons (the amount of salt per person one day is 6 grams), consuming nang with less salt (nang is a distinguishing foodstuff for the Uygur, which is baked with a salt surface) and eating less animal organs to control fat intake, eating more vegetables and other health education measures, and a combination of low-dose simvastatin drug intervention, explained that targeted measures taken to lipid-lowering therapy patients achieved a good target.

The control rates of dyslipidemia are the important indicators of evaluating the effective prevention of dyslipidemia. Li *et al.* reported that control rates of dyslipidemia was 3.53% among 197,409 subjects aged over 18 from 162 monitoring sites around 31 provinces in China mainland in 2010 [20]. It is revealed that the control rate of dyslipidemia have been comparatively low among Chinese adults, especially among the population who were young, or who were from rural areas or western China. A cardiovascular risk survey showed that a total of 4767 Uyghur were randomly selected from 26 villages in seven cities from the general population in Xinjiang and the control rate for dyslipidemia was 16.2% [16]. Those studies showed that the dyslipidemia control rate is very low in China. Our study also showed that using performed regular health education and provided lipid-lowering drugs could effectively adjust the lipid levels for local dyslipidemia patients.

After a three-year comprehensive intervention combined health education with drugs, it can effectively improve the level of knowledge, attitudes of dyslipidemia patients, further promote the transformation of patient behavior and improve confidence in treatment. Lai *et al.* reported that in a multi-center, 8-week, double-blind study, 304 adults who had received 8 weeks of stable statin therapy and found that the percent change from baseline in TG, TC, HDL-C and LDL-C were −4.87%, −3.13%, 0.10% and 8.16%, respectively [21]. Other studies reported evaluated 900 adults in a three-year intervention by primary care physicians, and the percent changes from baseline in TG, TC, HDL-C and LDL-C were −7.4%, −24.1%, 6.1% and −34.3%, respectively [22]. In summary, it is indicated that the longer the intervention period, the better the intervention efficiency, and the long-term sustainability of the standardized management can effectively control blood lipid levels.

This study showed that low-dose simvastatin and health education could significantly reduce blood lipid levels, also improved lipid control rate, and methods of taking statins were simple, inexpensive and acceptable. It is seen that taking a comprehensive prevention approach to prevent lipid level abnormalities among minorities in the remote rural areas of Xinjiang prevented and controlled the development of this chronic non-communicable disease in China's ethnic minority areas. Through three-years comprehensive interventions to strengthen health education combined with drug improved the lipid control rate of dyslipidemia patients, but its control was still low, and management of chronic diseases such as dyslipidemia also lack of effective and comprehensive prevention and multi-level management models. If a healthy lifestyle guidance taken earlier for the entire population, aroused enough attention from the government, who could intervene by providing stable investment, it will greatly reduce the potential harm to the patient’s family and community, reduce the social burden and workforce losses, so the prevention and control of dyslipidemia and its complications has important significance.

## 5. Conclusions

Using cheap comprehensive intervention and acceptable measures for minority dyslipidemia patients can be an effective lipid-lowering therapy in remote rural Xinjiang. Our comments and suggestions over the making of appropriate policies in public health would not only benefit low-income populations but also help middle-income individuals choose suitable strategies for disease prevention.
